# Age of diagnosis with endometriosis and potential predicting factors: A single-center cross-sectional study

**DOI:** 10.1371/journal.pone.0347136

**Published:** 2026-04-15

**Authors:** Soha Wahab, Zeina Chehade, Dima El Oueichak, Miguel Luna Russo, Miran A. Jaffa

**Affiliations:** 1 Faculty of Medicine, American University of Beirut, Beirut, Lebanon; 2 Institute for Women’s Health and Body, Complex Gynecology and Surgery, West Palm Beach, Florida, United States of America; 3 Epidemiology and Population Health Department, Faculty of Health Sciences, American University of Beirut, Beirut, Lebanon; Medical Park Minatomirai, JAPAN

## Abstract

**Objective:**

To evaluate the behavioral and clinical characteristics of endometriosis patients at the largest tertiary healthcare center in Lebanon and to analyze the effect of endometriosis symptoms, age of menarche, sexual activity, and infertility on the age of diagnosis with endometriosis.

**Design:**

Retrospective cross-sectional study of patients who presented to a tertiary healthcare center over 1 year (2018–2019) and were diagnosed with endometriosis (**N = 688**).

**Methods:**

A **symptom severity score (SSS)** ranging from 0 to 3 was employed to estimate the severity of symptoms, specifically focusing on chronic pelvic pain (CPP), dyspareunia, and neuropathic pain. We performed one-way analysis of variance (**ANOVA**), followed by Post Hoc analysis for multiple comparisons using the Least Significant Difference (**LSD**) method to determine the difference in age at diagnosis between groups presenting with different **SSS** values. We then conducted a **multiple linear regression analysis** to model the age of diagnosis with endometriosis as a function of severity of symptoms while adjusting for age of menarche, sexual activity, and infertility.

**Results:**

The mean age of diagnosis was **29.67** years, and the mean duration of follow-up was **4.74** years. The most common symptom was dysmenorrhea (75.7%). One-way analysis of variance (ANOVA) demonstrated a significant difference in the age of diagnosis among patients with different **SSS** values (**p = 0.006**). In the multiple linear regression analysis model, the age at diagnosis was earlier for patients with more severe symptoms compared to none, yet significance was present for **SSS = 1 (p ≤ 0.001)** and **SSS = 2 (p = 0.01)**, not for patients with the most severe presentation having **SSS = 3 (p = 0.23)**. This analysis further revealed that infertile patients had an earlier diagnosis of **2.72** years on average compared to fertile patients **(p ≤ 0.001).** Furthermore, age of menarche didn’t have a significant effect on the age of diagnosis **(p = 0.73).**

**Conclusion:**

Our results suggest that the severity of pain symptoms is not always indicative of an earlier diagnosis, which underscores the crucial need to enhance awareness regarding the clinical presentation of this condition in both society and the medical field.

## Introduction

Endometriosis is a prevalent disease affecting 10–15% of reproductive-age female patients, with an average delay in diagnosis of 8–12 years [1]. It is defined as a systemic inflammatory disease caused by the presence of endometrial-like tissue in extra-uterine sites [2]. Several risk factors have been associated with endometriosis, including early age at menarche and a family history of the disease [1,3]. Tamoxifen, which acts as an estrogen agonist in the endometrium, has also been reported in association with endometriosis, although its pathophysiology remains incompletely understood [4]. Due to diagnostic delays and lack of effective medical treatment options, research indicates that this disease represents a notable burden within the spectrum of benign gynecological conditions among patients in their reproductive years [5]. Clinically, endometriosis can manifest through a range of symptoms. Dysmenorrhea is the most reported symptom that suggests the presence of endometriosis, but many other manifestations exist including but not limited to urinary and bowel symptoms, dyspareunia, infertility, and chronic pelvic pain [6].

The existing literature on endometriosis has little generalizability to the Middle East & North Africa (MENA) population due to a huge knowledge gap relating to all aspects of this disease. A systematic review done by Moussa et al. estimated the prevalence of surgically confirmed endometriosis in the Middle East and Persian region to be 12.9% in women undergoing laparoscopy for any indication [7]. As for Lebanon, the most recent clinical study on patients with endometriosis was a retrospective case-control study of patient records at the American University of Beirut Medical Center (AUBMC) between 1979 and 1981 showing that this disease represented 2% and 21% of all gynecological cases and laparoscopic cases respectively [8].

In the current landscape, a singular large-scale case-control study conducted in the United Arab Emirates (UAE) stands out as it provides a detailed account of symptoms among 518 Arab endometriosis patients. However, it is noteworthy that this study does not encompass certain endometriosis-related symptoms, including dyspareunia, as well as urinary and bowel symptoms unrelated to dysuria and dyschezia respectively. Notably, their investigation reveals an average diagnostic delay of 11.61 years, a figure that escalates to 15.81 years in females who are not married, but no further investigation is done to evaluate for predictors of diagnostic delay [9]. This disparity may reflect sociocultural barriers to seeking gynecologic care, particularly among unmarried women, and hesitancy to seek evaluation for menstrual or pelvic symptoms.

Our study aims to characterize the behavioral and clinical features of patients with endometriosis at the largest tertiary healthcare center in Lebanon, the American University of Beirut Medical Center (AUBMC), and to identify predictors of age at diagnosis. Specifically, we examine the impact of symptom severity, age at menarche, sexual activity, and infertility on the age of diagnosis. Given the scarcity of regional data and the persistent global challenge of diagnostic delay, identifying these predictors is essential for informing strategies to improve patient care and reduce delays in diagnosis.

## Materials and methods

### Study design and target population

In this retrospective cross-sectional study, we employed convenience sampling; we included patients of all ages diagnosed with or treated for endometriosis who presented to AUBMC over one year between 01/11/2018 and 31/10/2019, with medical records indicating a suspected clinical diagnosis of endometriosis or a confirmed endometriosis diagnosis based on imaging or surgical findings.

Patients with abdominal wall endometriosis related to prior cesarean section without evidence of pelvic disease, as well as patients initially suspected to have endometriosis but subsequently found to have no evidence of disease on imaging or surgery, were excluded. After applying these criteria, a total of 688 patients were included; of these, 17 patients were diagnosed based on documented clinical pain symptoms particularly dysmenorrhea, while the remaining patients had confirmation through imaging, surgery, or both.

As the largest tertiary center in Lebanon, AUBMC receives patients from across the country, enhancing the representativeness of our sample for the general Lebanese population. The combination of convenience sampling from a diverse patient population and the application of clearly defined exclusion criteria helped minimize selection bias and support the generalizability of our findings.

### Variables of the study

All variables were tabulated and presented depending on their scale: categorical variables were summarized using frequencies and percentages; while continuous variables were summarized in terms of means, medians, range, and standard deviations (SD). Missing data were present only in continuous variables and ranged from 6% to 9% across variables. Given the relatively low proportion of missing values, missing observations were replaced with the mean of the corresponding variable calculated from available cases to retain the full analytic sample.

To address the research questions, we extracted data on behavioral and clinical characteristics of endometriosis patients, as well as potential predictors of age of endometriosis diagnosis. Table 1 presents behavioral characteristics including smoking, alcohol intake, sexual activity, in additional to age of menarche, age at diagnosis with endometriosis and follow-up period. Table 2 shows a detailed distribution of endometriosis-related symptoms among patients, as documented in gynecologist clinical notes at the time of patient presentation. Chronic Pelvic Pain (CPP) is defined as the presence of pain in the pelvis outside of menstruation for at least 6 months [10], while neuropathic pain is caused by a lesion or disease of the somatosensory system [11]. Symptoms of neuropathic pain may be variable, described in our population as burning or shooting pain often radiating to the legs or groin based on clinical documentation in the medical records. Infertility is defined as the inability to conceive after one year of trying for individuals under 35 or six months for those over 35. In addition to the symptoms, Table 2 also displays the number of patients referred to a pain center for pain symptoms, and the number of ED admissions at AUBMC due to endometriosis-related manifestations. Due to the absence of a validated scoring system for symptom severity, we utilized a **Symptom Severity Score (SSS)** ranging from 0 to 3, as detailed in Table 3. This composite score is a simplified measure reflecting the number of key pain-related symptoms - chronic pelvic pain (CPP), dyspareunia, and neuropathic pain – that impact patients’ quality of life, rather than the intensity or severity of each symptom. It is important to note that patients with an SSS of 0 may still have other endometriosis-related symptoms not captured within this score.

**Table 1:** Characteristics of endometriosis patients at a tertiary health care facility (N = 688 patients).

	n (%)	Mean (SD), median, range
**Age in years**		38.42 (8.47); 38; [20-72]
**BMI (in kg/m**^**2**^)		
<18.5 (Underweight)	28 (4.1)	
[18.5, 25[(Healthy)	470 (68.3)	
[25, 30[(Overweight)	138 (20.1)	
≥ 30 (Obese)	52 (7.6)	
**Smoking**		
Never smoked	383 (55.7)	
Ever smoked	305 (44.3)	
**Alcohol**		
Never used	434 (63.1)	
Ever used	254 (36.9)	
**Sexual Activity**		
Never Active	155 (22.5)	
Ever active	533 (77.5)	
**Age of menarche**		12.25 (1.03); 12.25; [8-17]
**Age at diagnosis with endometriosis**		29.67 (6.23); 29.67; [14-52]
**Average time for follow up (years)**		4.74 (5.13); 3; [1-28]

**Table 2:** Clinical presentation of endometriosis patients at a tertiary health care facility (N = 688 patients).

	n (%)
**Dysmenorrhea**	521 (75.7)
**Dyspareunia**	
Present	173 (25.1)
Not sexually active	155 (22.5)
**Heavy bleeding during regular menstruations**	178 (25.9)
**Spotting outside menstruation periods**	94 (13.7)
**Chronic Pelvic Pain (CPP) / Acyclical pain**	334 (48.5)
**Lower Back Pain**	79 (11.5)
**Neuropathic Pain** ^a^	60 (8.7)
**Gastrointestinal symptoms** ^b^	237 (34.4)
**Urinary symptoms** ^c^	132 (19.2)
**Fertility status**	
Fertile	144 (20.9)
Infertility	279 (40.6)
Not sexually active	155 (22.5)
Not actively trying to become pregnant	110 (16.0)
**Referral to a pain center**	2 (0.3)
	**Mean (SD), median, range**
**Number of admissions to the ER due to endometriosis symptoms (n = 164)** ^d^	2.3 (2.5); 1; [1-18]

^a^Neuropathic pain: present in the records or described as burning or shooting pain, radiating to the legs or groin.

^b^Gastrointestinal symptoms include bloating, IBS-like symptoms, pain during defecation, blood in stools, and tenesmus.

^c^Urinary Symptoms include recurrent urinary tract infections, urinary frequency & urgency, and dysuria.

^d^164 (23.8%) were admitted to ER at AUBMC for endometriosis symptoms.

**Table 3:** Frequency and percentage distribution of Symptom Severity Scores (SSS) among endometriosis patients (N = 688 patients).

SSS^a^	Frequency	Percentage (%)
**0**	**311**	**45.2**
**1**	**213**	**31**
**2**	**138**	**20.1**
**3**	**26**	**3.8**
**Total**	**688**	**100**

^a^SSS (Symptom Severity Score) = CPP (Chronic Pelvic Pain) + Dyspareunia + Neuropathic Pain; Range [0–3].

### Statistical analysis & Ethical approval

This retrospective cross-sectional study received IRB approval on January 17, 2022. The IRB waived consent since the research project was classified as “minimal risk.” No patient was contacted. Between 17/01/2022 and 31/08/2023, data was extracted from the EPIC EMR database at AUBMC, implemented in November 2018. The study investigators de-identified the data before its inclusion in the database.

To determine the difference in age of diagnosis with endometriosis between subgroups of endometriosis patients having different severity of symptoms as per the **SSS**, we performed one-way analysis of variance (**ANOVA**), followed by Post Hoc analysis for multiple comparisons using the Least Significant Difference (**LSD**) method (Table 4).

**Table 4:** Comparison of the mean age of diagnosis among endometriosis patients based on the severity of the symptoms (N = 688 patients).

ANOVA Test				
Between Groups	p-value^a^ = 0.006			
Post Hoc Test using LSD^b^				
SSS^c^(I)	SSS (J)	Mean Difference in Age of Diagnosis (I-J)	P-value	95% Confidence Interval
**0**	**1**	**1.87**	**0.001**	[0.79 - 2.95]
**0**	**2**	0.51	0.42	[-0.73 - 1.76]
**0**	**3**	−0.33	0.8	[-2.8 - 2.16]
**1**	**2**	**−1.36**	**0.045**	[-2.69 - -0.3]
**1**	**3**	−2.2	0.08	[-4.72 - 0.33]
**2**	**3**	−0.84	0.52	[-3.44 - 1.76]

^a^p-value ≤ 0.05 is considered significant.

^b^LSD: Least Significant Difference for multiple comparison testing

^c^SSS (Symptom Severity Score) = CPP (Chronic Pelvic Pain) + Dyspareunia + Neuropathic Pain; Range [0–3].

We then conducted **multiple linear regression analysis** to determine the adjusted association between several predictors that included severity of symptoms, age of menarche, sexual activity, and infertility with the age of diagnosis as the outcome of interest (Table 5). Given that endometriosis is classified as a gynecological disease and considering the substantial focus on fertility within our population, we accounted for factors that influence when patients seek medical assistance for endometriosis symptoms to consider their impact on the age of diagnosis.

**Table 5:** Comparison of the mean age of diagnosis of endometriosis patients based on the severity of the symptoms using multiple linear regression analysis (N = 688 patients).

Age at Diagnosis	Coefficient	P-value^a^	95% Confidence Interval
**SSS**^b^ = 1	−2.12	**< 0.001**	[-3.2 - -1.05]
**SSS = 2**	−1.72	**< 0.001**	[-2.97 - -0.48]
**SSS = 3**	−1.50	0.23	[-3.94 - 0.93]
**Age of Menarche**	0.01	0.73	[-0.06 - 0.08]
**Sexual Activity**	4.74	**< 0.001**	[3.50 - 5.97]
**Infertility**	−2.72	**< 0.001**	[-3.78 - -1.65]

^a^p-value ≤ 0.05 is considered significant.

^b^SSS = Symptom Severity Score.

The level of significance was assumed to be ≤ 0.05. All analysis was conducted using **STATA version 17**.

## Results

As shown in Table 1, the mean age of the 688 patients included in the study was 38.42 years (SD = 8.47) and the range was 20–72 years. A healthy BMI was observed in 68.3% of patients; 55.7% reported never smoking, and 63.1% reported no alcohol use. The mean age at menarche was 12.25 years (SD = 1.03), and 77.5% of patients had engaged in sexual activity prior to diagnosis. The mean age at endometriosis diagnosis was 29.67 years (SD = 6.23) with a range of 14–52 years, and the mean follow-up duration was 4.74 years with a range of [1–28] years.

Table 2 presents the prevalence of each endometriosis symptom in our cohort. Dysmenorrhea was reported in 75.7% of patients, while chronic pelvic pain or acyclical pain was reported in 48.5%. Dyspareunia was reported in 25.1% of patients, and neuropathic pain was the least prevalent of all symptoms (8.7%). Furthermore, only 2 of the 688 patients were referred to a pain center to manage their symptoms, and 164 patients were admitted to the ER due to endometriosis symptoms, with a mean of 2.3 admissions per patient. Heavy bleeding was documented in the charts of 178 patients (25.9%), out of whom 97 (54.5%) had a diagnosis of fibroids or adenomyosis. Lastly, 279 out of 688 patients (40.6%) experienced infertility, accounting for 65.9% of those actively trying to conceive.

In Table 3, we divided patients into 4 groups based on the severity of symptoms determined by **SSS**. **45.2%** of patients had none of the three severe symptoms, **31%** presented with SSS = 1, **20.1%** had SSS = 2, while **3.8%** had all three symptoms considered severe.

In Table 4, the **one-way ANOVA test** shows an overall significant difference in the age of diagnosis among the 4 SSS groups with **p-value = 0.006**. Post Hoc testing for multiple comparisons via the **LSD method** showed a significant pairwise difference between **SSS = 0** & **SSS = 1**, and between **SSS = 1** & **SSS = 2**. In particular, patients with 1 severe symptom were diagnosed earlier than patients presenting with no severe symptoms by **1.87 years** (**p-value = 0.001**), while patients with 2 severe symptoms were diagnosed later by **1.36 years** compared to patients with 1 severe symptom (**p-value = 0.045**).

Table 5 summarizes the results of the **multiple linear regression analysis** performed to examine the association between the value of **SSS** and the mean age of diagnosis, while adjusting for age of menarche, sexual activity, and infertility as covariates. A trend can be seen where patients presenting with more severe symptoms are diagnosed earlier than those presenting with no severe symptoms, yet this is only significant for patients with **SSS = 1** diagnosed earlier by **2.12 years (p < 0.001)**, and for patients whose **SSS = 2** diagnosed **1.72 years** earlier compared to patients with no severe symptoms **(p < 0.001)**. To note, patients with the most severe presentation (**SSS = 3**) had an earlier diagnosis by one and half years but this result was not significant. In addition to that, sexually active patients had a delayed diagnosis of **4.74 years (p < 0.001)** compared to non-sexually active patients, while patients with infertility were diagnosed earlier by **2.72 years (p < 0.001)** compared to others, when controlling for the severity of the symptoms.

## Discussion

In this study, dysmenorrhea and chronic pelvic pain were the most common symptoms. Most patients had no or only one severe symptom based on the symptom severity score. Greater symptom severity tended to be associated with earlier diagnosis, although this was not consistent across all groups. In adjusted analyses, infertility was associated with earlier diagnosis, whereas sexual activity was associated with delayed diagnosis.

In concordance with other studies, most of our patients experienced dysmenorrhea (9, 12–14), and almost half reported CPP, yet less than 1% were referred to the pain center available at AUBMC. Gastrointestinal symptoms, including bloating, dyschezia and hematochezia were present in 34.4% of patients. Among individuals reporting heavy menstrual bleeding, 54.7% were diagnosed with adenomyosis or fibroids, conditions recognized for causing this symptom. This finding implies a potential overestimation in attributing heavy bleeding to endometriosis in clinical practice.

While the delay in diagnosis could not be determined due to the lack of information on the age at which the first symptom appeared, the average age of endometriosis diagnosis in our cohort of patients was 29.67 years old. The demographic and clinical characteristics of our cohort (Table 1) also provide context for the diagnosis of endometriosis. Previous studies suggest that certain patient characteristics may influence disease risk and recognition; for example, higher BMI has been associated with longer diagnostic delay, while earlier menarche has been linked to an increased likelihood of developing endometriosis [1].

In a cross-sectional study involving 512 Arab patients conducted in the **United Arab Emirates (UAE)**, the average age of initial symptom onset for endometriosis was found to be 17.1 years [9]. Consistent findings emerged from a prospective French study that encompassed 57 endometriosis patients, revealing an average onset of symptoms at 16 years old [12]. In a cross-sectional study involving over 4,334 patients diagnosed surgically with endometriosis, more than two-thirds of respondents reported symptoms onset during adolescence [13]. Similar ages at symptom onset have been reported in other populations, including studies from New Zealand and Italy [14,15]. Taken together, these observations suggest that the interval between symptom onset and the age at diagnosis observed in our cohort may approach a decade.

Research looking into the causes of the delayed diagnosis is limited. Devenport et al. conducted a systematic review to explore barriers to early endometriosis diagnosis from the perspectives of both the patients and general healthcare practitioners. Several themes were established, including the normalization of period pain (dysmenorrhea) in the society, and the tendency to label patients as exaggerators, both of which deter patients from seeking timely medical attention [16]. On the other hand, general healthcare professionals reported lack of adequate clinical training and knowledge on this condition, and inability to decide with confidence when a referral to a specialist must be done, given the variability of endometriosis symptoms. Another concerning issue is the dismissal of endometriosis based on negative imaging results [16]. Non-invasive diagnostic imaging falls short in ruling out superficial endometriosis, and identifying deep endometriosis through MRI or transvaginal ultrasound demands a high level of expertise, which does not sufficiently meet the demand of the public [17,18]. Furthermore, medical gaslighting emerges as a significant concern reported by patients seeking help, reflecting a situation where their symptoms are not taken seriously enough to prompt further investigation [16]. The lack of a significant earlier diagnosis for patients presenting with **SSS = 3** compared to those with **SSS = 0** raises questions about the perception of patients presenting with multiple severe symptoms in the setting of insufficient clinical training in this field. It suggests that patients reporting many symptoms, particularly severe symptoms, may be subject to delayed diagnosis due to gaslighting. Nevertheless, as we lack information on when patients sought medical attention, our data does not provide substantial support for this hypothesis.

As expected, patients with infertility were diagnosed earlier by 2.72 year compared to others while adjusting for the severity of the symptoms. In fact, 65.9% of patients actively trying to become pregnant suffered from infertility. The earlier diagnostic age in this subgroup could be partially ascribed to the societal emphasis on fertility, leading couples to proactively seek medical assistance at an early stage when facing challenges in conception [19].

Finally, sexually active patients had a delayed diagnosis of 4.74 years compared to non-sexually active patients when adjusting for the severity of the symptoms. Given the prevalence of religious beliefs and societal norms discouraging premarital sexual relationships, the commencement of sexual activity is anticipated to occur at a later age in the Lebanese society, after which patients may seek medical attention for symptoms related to the cycle or sexual intercourse leading to the diagnosis of endometriosis. These findings highlight the important role of sociocultural factors in shaping access to gynecologic care and, consequently, diagnostic timelines. In settings where cultural norms discourage open discussion of menstrual or sexual health, patients may delay seeking medical attention for symptoms such as pelvic pain or dyspareunia. This delay in care-seeking behavior, compounded by potential provider-related barriers and variability in clinical suspicion, may contribute to prolonged diagnostic delay in endometriosis.

### Strengths & limitations

Several limitations were identified in this study. Firstly, data extraction relied on healthcare provider notes documented in the EPIC Database. In addition, the classification of symptoms, including neuropathic pain was based on physician documentation rather than a validated assessment tool, which may have introduced misclassification bias. Moreover, the presence of undiagnosed concurrent comorbidities, particularly in older patients, including pelvic floor dysfunction and musculoskeletal disorders cannot be excluded and may have contributed to the reported symptoms. Finally, the absence of information about the age of symptom onset hinders accurate diagnostic delay determination. Nevertheless, existing literature provides relevant data to make an approximate inference in this regard.

This study’s strengths reside in its examination of the largest cohort of endometriosis patients in the MENA region. It offers a thorough depiction of both behavioral and clinical characteristics and fills a gap in the literature by assessing predictors of age at diagnosis with endometriosis, an aspect often overlooked in existing research.

## Conclusions

Endometriosis is a systemic disease characterized by a range of symptoms that healthcare professionals should be knowledgeable about to foster accurate suspicion. Patients with endometriosis who present with infertility and up to two severe symptoms tend to receive an earlier diagnosis than those with no symptoms. However, the age of diagnosis is not significantly different for patients who report all three endometriosis pain symptoms (CPP, Dyspareunia, and Neuropathic pain). These study findings suggest that the severity of symptoms is not always a reliable indicator of early diagnosis, highlighting the need for increased awareness of the clinical presentation of this condition among both the public and healthcare professionals. By doing so, we can reduce delays in diagnosis and facilitate earlier treatment for the condition.
